# Thrombus perviousness in acute ischemic stroke: a scoping review of methodology, predictive value, and future perspectives

**DOI:** 10.1007/s00234-025-03627-9

**Published:** 2025-04-24

**Authors:** Gergely Bertalan, Patrick Thurner, Jawid Madjidyar, Miklós Krepuska, Vania Anagnostakou, Anna Kyselyova, Tilman Schubert, Zsolt Kulcsar

**Affiliations:** University Hospital of Zurich, Zurich, Switzerland

**Keywords:** Acute ischemic stroke, Computed tomography, Thrombus perviousness, Dynamic perviousness, Clinical outcome, Mechanical thrombectomy, Thrombus structure

## Abstract

**Purpose:**

Thrombus perviousness, depicting the interaction of occlusive clot with contrast media as measured with computed tomography (CT) is a relatively new imaging biomarker in acute ischemic stroke (AIS). This approach holds the potential to tailor revascularization strategies and post-interventional treatments, thereby enhancing functional outcomes. However, its predictive value is not yet conclusive despite its association with several clinical parameters.

**Methods:**

This scoping review provides a comprehensive overview of 51 articles that explore this issue. It focuses on the analysis of applied methodology in measuring perviousness, the predictive value of perviousness based on available data, and the future perspectives and potentials this biomarker may have in AIS imaging.

**Results:**

Although some data are contradictory, in the vast majority of published studies, pervious thrombi were easier to remove with mechanical thrombectomy, responded better to thrombolytic treatment and showed better functional outcome than impervious thrombi. The methodology of measuring perviousness is not yet standardized, which may lead to inconsistency in the findings. New data on time-resolved (dynamic) perviousness show more promising results and refined characterization of occlusive thrombi in AIS.

## Introduction

Each year, approximately 77.2 million people worldwide experience a stroke, with 63.5 million facing short- or long-term disability and 3.3 million not surviving [1]. Over 85% of cases are acute ischemic strokes (AIS) caused by an obstructive thrombus [2]. Mechanical thrombectomy (MT), combined or not with systemic thrombolysis, is the gold standard for AIS treatment with large vessel occlusion [3]. In this endovascular procedure, the thrombus is removed using a stent-retriever, direct aspiration with a large lumen catheter, or both, aiming for rapid and complete revascularization, which is associated with good clinical outcomes and lower mortality [4, 5]. However, in approximately 10–50% of cases, substantial revascularization is not achieved, resulting in unsatisfactory clinical outcome [6]. While the reason for this is multifaceted, it is believed that thrombus composition and mechanical properties are key factors, leading to efforts to predict these properties from pre-interventional imaging.

Histological analyses of human thrombi in AIS have shown that thrombi mainly consist of fibrin, platelets, and red blood cells (RBCs), and can contain smaller amounts of white blood cells (WBCs), lipids, von Willebrand factor, neutrophil extracellular traps, calcification and extracellular DNA [7–10]. It has been reported that recanalization was less successful for thrombi, which are rich in platelets or DNA and tend to be stiff [9, 11], while relatively hard and fibrin-rich thrombi were associated with longer intervention times [12, 13]. On the other hand, intervention times for soft, RBC-rich clots were shorter and a smaller number of passes was required [14–16]. These studies underline the importance of thrombus composition in the treatment of AIS. However, neither state-of-the-art methods for in vivo structure estimation nor established guidelines for selecting an MT approach based on thrombus structure exist.

Neuro-vascular imaging is the cornerstone for the diagnosis and management of AIS, by providing information on the salvageable and infarcted brain tissue, location of vessel occlusion, and thrombus physical characteristics such as length, volume or shape. All these factors will define the revascularization strategy and help to select the tools and techniques of MT. Computed tomography (CT) is the gold standard in AIS diagnosis to make time-critical decisions due to its availability in emergency departments and fast acquisition times. Guided by the principle of “time is brain”, imaging must rapidly and accurately diagnose AIS, assess the damaged and yet salvageable brain tissue and depict the vessel occlusion — all within the shortest possible time frame. In the acute settings of AIS, conducting a detailed and time-intensive image analysis of an occlusive thrombus would not be ethical. As a result, we currently depend on the information available from routine imaging scans.

In non-contrast CT (NCCT) images, clots may appear as hyper-attenuated regions, known as the hyperdense artery sign (HAS) [17]. After intravenous administration of a contrast material (CM), in CT angiography (CTA), blood vessels are contrast-enhanced, while an occlusive, impermeable thrombus appears as a lack of signal. CTA allows for the evaluation of clot burden, collateral circulation and clot length if distal perfusion is sufficient [17]. CT perfusion (CTP) quantifies the blood flow in the brain using CM followed by a series of rapidly acquired images. CTP allows for the differentiation between tissue that is already infarcted (irreversibly damaged) and tissue that is at risk but still salvageable (penumbra) [17].

Many studies have focused on CT signal intensity measured as Hounsfiled Unit (HU) or the presence of the HAS on NCCT. HAS is associated with a higher content of RBCs [18–20] and calcification [21, 22] and is often absent in platelet-rich thrombi [22]. Shin et al. [19] found that RBC-rich clots are associated with HAS and successful recanalization, while Brinjikji et al. [23] reported 45% and 25% mean RBC in thrombi with and without HAS, respectively. However, other studies suggested that higher HU values were linked to increased fibrin content rather than RBCs, as well as longer intervention times [24], or found no correlation between HU values, recanalization success [20, 25], or RBC content [20]. Therefore, the quest for an imaging biomarker for thrombus characterization remains ongoing.

Thrombus perviousness, as measured with CT, was recently introduced for blood clot permeability estimation [26]. Using standard CT images (NCCT, CTA, CTP), perviousness quantifies the increase in CT attenuation caused by the CM uptake of the clot. Since CM uptake in thrombi is influenced by various physical factors like thrombus location, shape, density, porosity, and histological composition [21, 27], several studies have explored the role of thrombus perviousness in AIS, examining its relationship with reperfusion parameters, clinical outcomes and histopathological composition. If the mechanical and/or structural characteristics of the thrombus can assessed through perviousness, the technical removal concept could be adapted to the expected recanalization difficulty. However, to incorporate perviousness into the clinical practice of AIS, two key questions must be addressed. First, whether perviousness is linked to clinical parameters, including revascularization parameters and thrombus structure. If such a correlation is established, the next question is how imaging can be used to effectively characterize perviousness. While Cahalane et al. [21] provided a brief review of six articles examining thrombus perviousness in relation to structural properties, a more in-depth review of the current literature from a neuroradiological standpoint is still needed. Therefore, this scoping review seeks to summarize research on thrombus perviousness in AIS within the clinical context.

## Literature search

The literature search was conducted in PubMed to identify publications addressing thrombus perviousness in AIS. Although thrombus perviousness is a proxy for thrombus permeability and the two methods are closely related, this review focuses only on thrombus perviousness as measured with CT. The search criteria in PubMed were as follows: *thrombus perviousness* OR *perviousness stroke*. No limit was imposed on the date of the publications. The search was conducted in October 2024 and resulted in 51 publications. Inclusion criteria were: 1) in vivo or ex vivo studies using human thrombi, 2) thrombus perviousness measured with CT and 3) studies that relate perviousness to either composition, mechanics or clinical parameters. After screening the abstracts and conclusions of the articles, two articles were excluded and 49 were included in this review. The included studies primarily focused on one or more of the following aspects: 1) methodology, 2) perviousness and histopathological clot structure, and/or 3) perviousness in relation to clinical parameters. Accordingly, the publications were summarized across these three categories. The scoping review was conducted following the PRISMA-ScR (Preferred Reporting Items for Systematic Reviews and Meta-Analyses extension for Scoping Reviews) guidelines [28]. Ethics declaration: not applicable.

## Measurement of thrombus perviousness with CT

In clinical and research settings, perviousness is sometimes used interchangeably with permeability, but their subtle differences can be important when describing the assessment procedures. While both terms deal with the ability of a thrombus to allow flow through it, permeability is a precise physical term used in fluid dynamics and requires measurements under controlled conditions (e.g. pressure-driven flow). In contrast, perviousness is a more descriptive and broader term used in the clinical and imaging contexts for emphasizing the overall ability of the thrombus to let substances pass through, including how"open"or"closed"the structure is.

Most clots will produce no or weak signals on NCCT, while their brightness on CT images after intravenous injection of a CM, such as CTA, is determined by how much contrast material they take up (high contrast uptake – high signal intensity). Perviousness quantifies the CT attenuation increase using either two or more than two imaging time points. Because the most widely used method in the literature is the version with two imaging time points, we will refer to these approaches as *standard* and *dynamic perviousness*, respectively.

The CT acquisition parameters in the clinical routine of AIS are scanner and provider-dependent with typical values as follows. NCCT: tube voltage 120–140 kVp, tube current 200–600 mA, in-plane resolution 0.5 × 0.5 mm^2^, slice thickness 0.5–5 mm, axial slices with a size of 512 × 512, 10–60 s acquisition time. CTA: tube voltage 120–140 kVp, tube current 150–300 mA, image resolution 0.5 × 0.5 × 0.5 mm^3^, axial slices with a size of 512 × 512, 10–60 s acquisition time, intravenous contrast medium (60–80 mL) with a rate of approximately 5 mL/s. A typical delay between NCCT and CTA is approximately 80 ± 30 s. CTP: tube voltage 70–120 kVp, tube current 150–300 mA, in-plane resolution of 0.5 × 0.5 mm^2^, slice thickness 1–4 mm, axial slices with a size of 512 × 512, 20–40 phases with 1–3 s cycle time, 100–200 s total acquisition time.

### Standard perviousness

The measurement of thrombus perviousness in the clinical routine is challenging and, therefore, two simplified measures have been proposed: 1) the thrombus attenuation increase (TAI) and 2) the void fraction method. Both methods will be described based on Santos et al. [26].

**Fig. 1:**
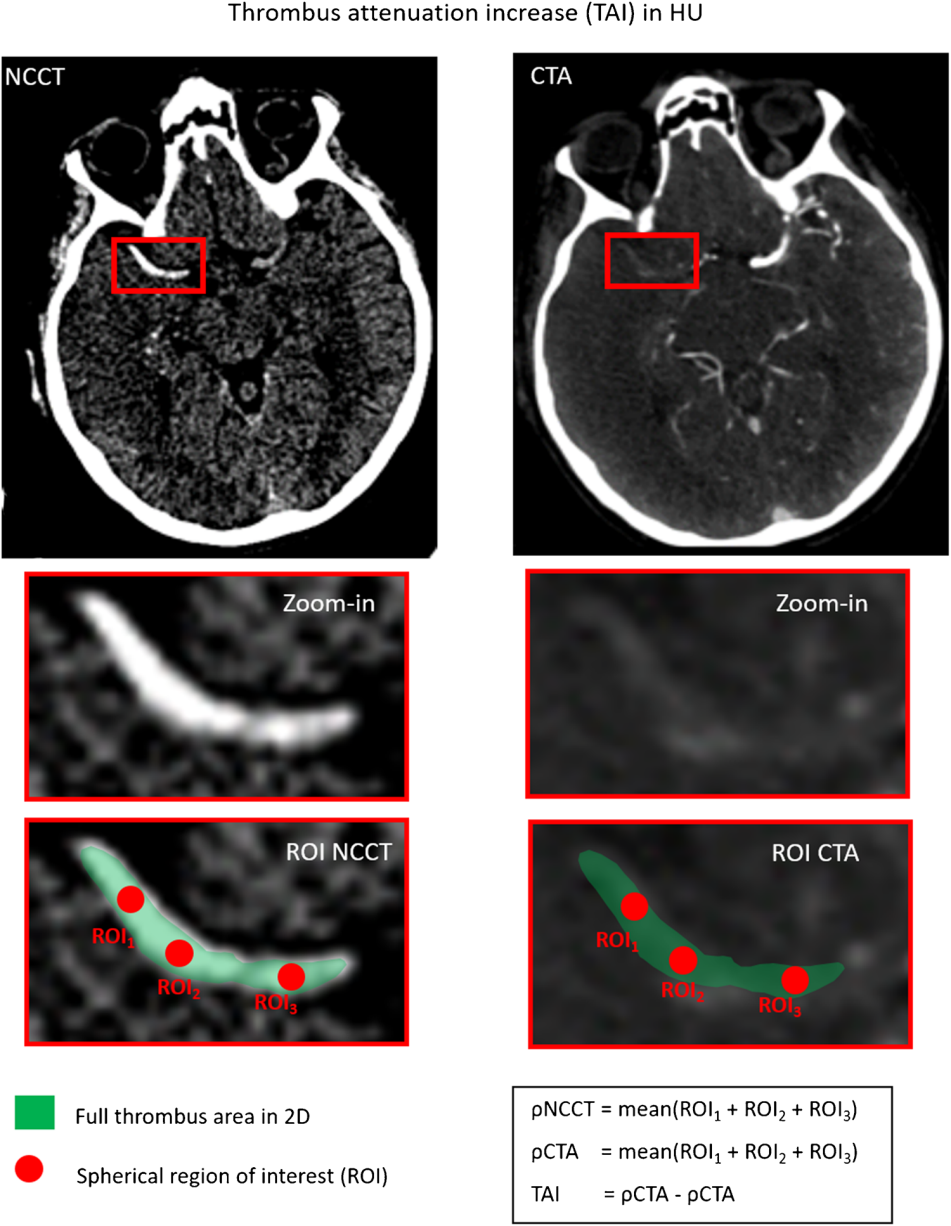
Calculation of thrombus perviousness as thrombus attenuation increase (TAI) between non-contrast CT (NCCT) and CT angiography (CTA). As this method is the most widely used in the literature, it is referred to as the standard perviousness (TAI_standard_) in this review

Figure 1 illustrates the calculation of TAI, which is the mean clot density difference between NCCT and CTA. In short, three spherical regions of interest (ROIs) with a diameter of 1–2 mm are manually placed on the clot, both on NCCT and CTA. The average of every three ROIs is calculated and used as ρNCCT and ρCTA (in Hounsfield Unit, HU):

1 $$\rho \text{NCCT}=mean({ROI}_{1}^{NCCT}+ {ROI}_{2}^{NCCT}+ {ROI}_{3}^{NCCT} )$$

2 $$\rho \text{CTA}=mean({ROI}_{1}^{CTA}+ {ROI}_{2}^{CTA}+ {ROI}_{3}^{CTA} )$$

Perviousness is then computed as TAI = ρCTA – ρNCCT.

The void fraction (ε) is calculated similarly but the TAI of the thrombus material is corrected with the contrast enhancement of blood, as follows. ε is the ratio between a void volume, which is filled with blood (V_Blood_) and the total volume of the thrombus (V_Thrombus_):

3 $$\upvarepsilon =\frac{{V}_{Blood}}{{V}_{Thrombus}}$$

The attenuation in the thrombus is approximated as the linear combination of the attenuation of thrombus material (ρ™) and blood (ρ^Blood^):

4 $${\uprho }^{Thrombus}=\left(1-\upvarepsilon \right){\uprho }^{TM}+{\varepsilon \rho}^{Blood}$$

Applying Eq. 4 on NCCT and CTA gives:

5 $${{\uprho }_{\text{NCCT}}}^{Thrombus}=\left(1-\upvarepsilon \right){{\uprho }_{\text{NCCT}}}^{TM}+{\upvarepsilon {\uprho }_{\text{NCCT}}}^{Blood}$$

6 $${{\uprho }_{\text{CTA}}}^{Thrombus}=\left(1-\upvarepsilon \right){{\uprho }_{\text{CTA}}}^{TM}+{\upvarepsilon {\uprho }_{\text{CTA}}}^{Blood}$$

The measured attenuation of thrombus material is independent of the CT modality as it is assumed that no CM can flow into a plain material. Therefore:

7 $${{\uprho }_{\text{NCCT}}}^{TM}={{\uprho }_{\text{CTA}}}^{TM} ={\uprho }^{TM}$$

ρ_NCCT_^Blood^ and ρ_CTA_^Blood^ are the intensities of blood on NCCT and CTA, which can be measured using ROI in the contralateral arteries. Subtracting Eq. 5 from 6 results in ${{\uprho }_{\text{NCCT}}}^{Thrombus}+{{\upvarepsilon {\uprho }_{\text{CTA}}}^{Blood}={\uprho }_{\text{CTA}}}^{Thrombus}+{\upvarepsilon {\uprho }_{\text{NCCT}}}^{Blood}$, which leads to:

8 $$\upvarepsilon =\frac{({{\uprho }_{\text{CTA}}}^{Thrombus} - {{\uprho }_{\text{NCCT}}}^{Thrombus})}{({{\uprho }_{\text{CTA}}}^{Blood}- {{\uprho }_{\text{NCCT}}}^{Blood})}=\frac{(\Delta )}{({\Delta }_{c})}$$

According to Eq. 8, perviousness is the attenuation increase ∆ in the thrombus between CTA and NCCT scans caused by CM penetration into the thrombus, which is then corrected with the enhancement of blood (∆_c_) in the contralateral artery.

Berndt et al. [29] proposed a CTA-index as a simplified measuring method for thrombus perviousness. The authors used 1.5 mm spherical ROIs to cover a section behind the occlusion side and a second ROI at the corresponding position of the contralateral artery on a single-phase CTA image. Mean HU was computed for both sides (occlusion: HU_T_; contralateral: HU_c_) and the relative thrombus attenuation was calculated using the following CTA-index:

9 $${CTA}_{index}=\frac{ {HU}_{T} - {HU}_{c}}{{HU}_{T}+ {HU}_{c}}$$

TAI, void fraction, and CTA index, assessed using two imaging time points (NCCT and CTA) are the conventional parameters for evaluating perviousness, with TAI being the most commonly used method. Although TAI is less accurate than the void fraction, it is widely accepted for assessing clot perviousness in clinical practice due to its faster determination and the fact that the contralateral artery may not always be fully visible because of oblique slice orientation. Since TAI is the standard approach in the literature for characterizing thrombus perviousness, we will refer to it as TAI_standard_ for the remainder of this manuscript.

In the context of TAI, a clot is regarded as pervious when the TAI exceeds 10 (i.e. it shows enhancement of more than 10 HU on CTA) [20, 30], indicating some degree of CM penetration into the clot. This implies that the thrombus is not entirely occlusive and may permit partial blood flow.

### Dynamic perviousness with 3-phase CTA

Perviousness may be underestimated using the standard approach with single-phase CTA due to timing constraints, hemodynamic limitations, or even pseudo-occlusion [31, 32]. It has been reported that the delayed phases following the arterial peak phase in dynamic CTA can offer improved thrombus visualization and prognostic insights, potentially influencing treatment decisions in the acute phase [31, 33], which motivated the use of 3-phase CTA for perviousness assessment.

Santos et al. [34] reported a modified version of TAI_standard_ in which multiphase CTA with four imaging time points was used (NCCT, arterial phase CT, venous phase CT and delayed phase CT). They computed TAI_arterial_ (between arterial and NCCT), TAI_venous_ (between venous and NCCT), TAI_delayed_ (between delayed and NCCT) and TAI_maxCTA_ (between the temporal maximum intensity projection of the three CTA phases and NCCT). However, the authors concluded that arterial phase CTA was superior to venous phase CTA (8 s after arterial phase CTA) or delayed-phase CTA (16 s after arterial phase CTA) to assess the TAI and dynamic CTA imaging had no additional benefit [34]. Recently, Bertalan et al. [35] used a similar approach and characterized perviousness using three imaging time points. They computed TAI between NCCT and CTA as well as the late uptake and early washout of CM between CTA and late venous phase CT (CTV). Contrary to Santos et al. [34], they found no correlation between TAI_standard_ (with NCCT and arterial phase CTA) and clinical parameters while the TAI between CTV and CTA showed a significant correlation underlying the importance of late CM uptake and washout in perviousness characterization. Chen et al. [36] reached a similar conclusion using CTP. The authors argued that 3-phase CTA did not cover enough time points, and the optimal phase could be located between the arterial and venous phases [36].

### Dynamic perviousness with multiple time points

**Fig. 2:**
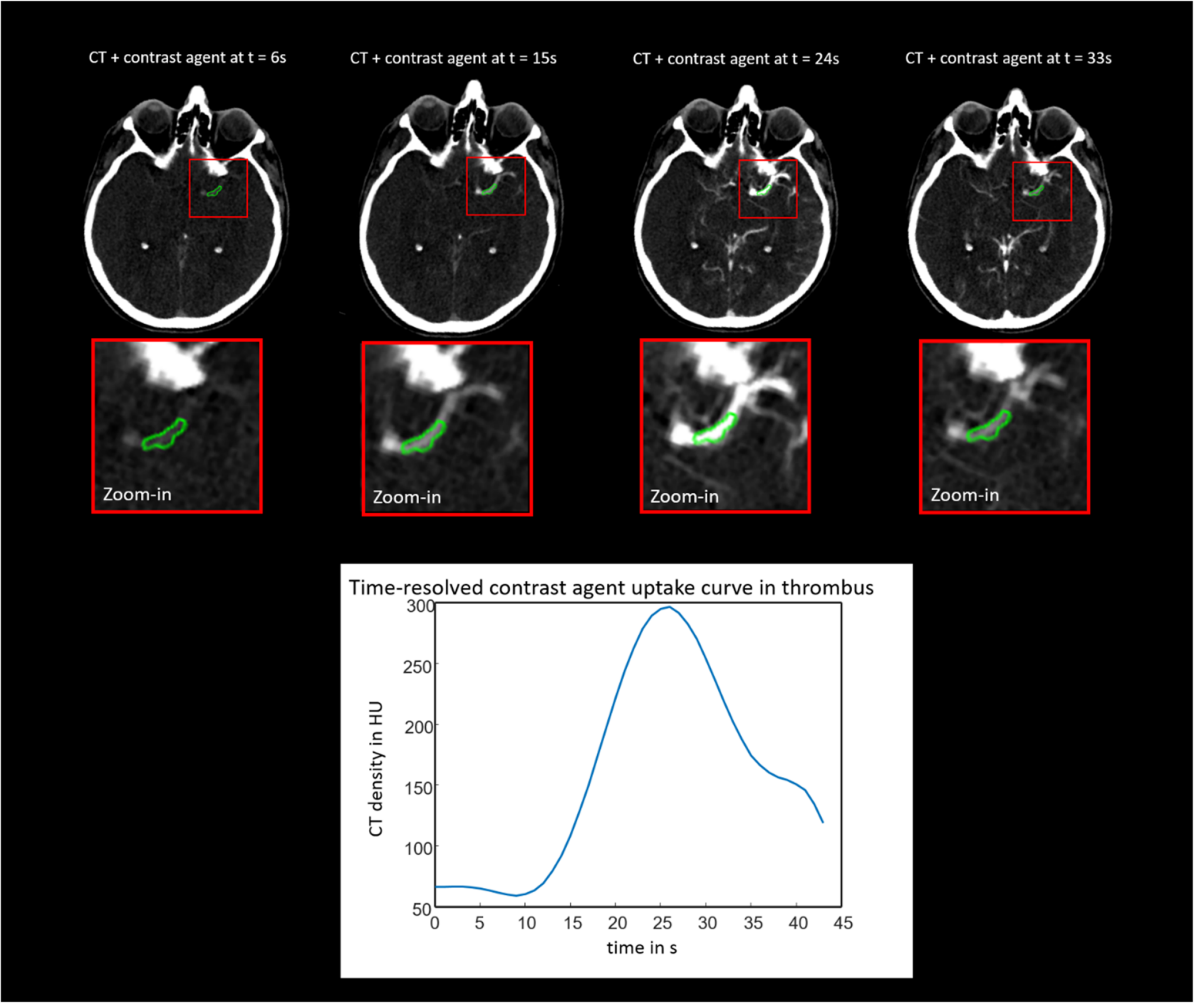
Time resolved contrast agent uptake curve (CAU) in a thrombus volume determined using the time-resolved imaging series of a CT perfusion scan. TAI_standard_ characterizes this contrast agent uptake curve with only two imaging time points, which can lead to substantial mischaracterization of thrombi through a signal processing phenomenon known in the literature as “under-sampling”. CTP parameters: 80 kV tube voltage, 3.0 mm slice thickness, 512 × 512 image size with 0.4 × 0.4 mm in-plane resolution, 30 phases with 1.5 s increments, 45 s total scan time

CM penetration into the clot is a dynamic process as illustrated with the time-resolved CM uptake curve (CAU) in Fig. 2. From the physical point of view, it is expected that the shape of the CAU (e.g. peak value, slope, width, etc.) is influenced by the clot composition and physical properties such as porosity, stiffness, shape, or location of the clot, and can show different patterns (e.g., fast uptake with early washout, late uptake with slow washout, no uptake, etc.). Consequently, various authors have been motivated to use the dynamic series of CTP or multiphase CTA for assessing perviousness.

Cheng et al. [36] measured perviousness assessed on a 26-phase dynamic CTA. The mean attenuation of thrombus on phase 1 was set as the baseline value and used to compute three TAI parameters: TAI_max_ (the maximum TAI among the 26 phases), TAI_peak_ (TAI between baseline and arterial peak phase) and TAI_con_ (TAI between baseline and peak on the normal side of the middle cerebral artery). Bertalan et al. [37] used a similar approach with the imaging series of a 30-phase CTP measurement. They derived time-dependent parameters of the CM uptake, such as the rise time and the TAI increase rate per second between the min and max values of the CAU or the time window for the 10, 20 and 30 percentile of CM peak concentration in thrombi. Wei et al. [38] measured and fitted the CAU either with a Gaussian (unimodal curve with one clear peak value) or with a linear model (linear curve without a clear peak value). The distribution of the CAU was used to predict structural composition or clinical parameters. All three groups came to the conclusion that methods using only a limited number of imaging time points to characterize the CM uptake can lead to substantial mischaracterization [36–38].

### The key factors that impact the evaluation of perviousness

**Fig. 3:**
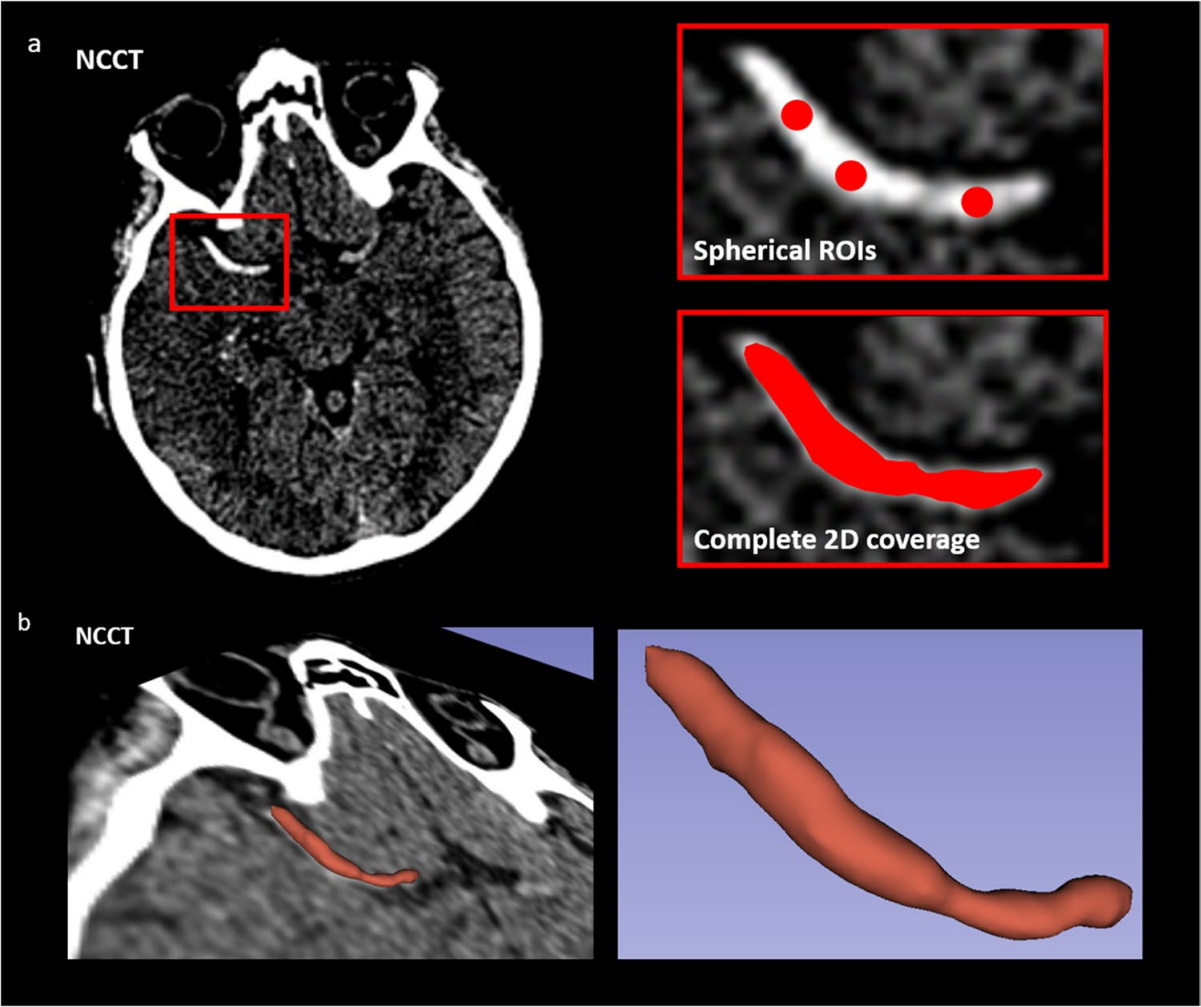
Thrombus segmentation in 2D (**a**) and in 3D (**b**). The vast majority of published studies used the version with three spherical ROIs in (a)

Thrombus segmentation for quantitative assessment remains one of the primary challenges in evaluating perviousness, regardless of the method used. The occluding clot may not always be visible on the NCCT, and its location is often only estimated based on the absence of enhancement on CTA, which indicates a disruption in blood flow in the blocked artery. As a result, clot segmentation is heavily reliant on the quality of the obtained images (e.g., motion artifacts, low image noise, etc.) and the expertise of the neuroradiological team. Figure 3 illustrates the main methods for thrombus segmentation. The majority of published studies relied on the 2D method with three spherical ROIs on a single transversal slice as shown in Fig. 3a. A potential disadvantage of this method is that the thrombus may not be completely covered, and the placement of the ROIs can introduce a significant bias in the computation. In Bertalan et al. [35], the HAS in the NCCT was covered with an ROI at each transversal slice position, resulting in a full 3D volumetric segmentation of thrombi (Fig. 3b). Although this method is more precise than the spherical ROI approach, determining the full volume of thrombi without HAS can be challenging as the precise spatial location of the clot can only be estimated based on the absence of signal in the CTA.

The next challenge is deciding between standard and dynamic assessment methods. Using only two imaging time points with an incorrectly chosen time delay for CTA acquisition can miss the peak concentration of the CM by either not permitting sufficient time for the CM to interact with the occlusive thrombus or by hitting a phase in which the CM is already washed out from the clot. Standard perviousness with two imaging time points can capture either the early, peak, or late phase of the CM concentration in the clot, but not all three simultaneously, which can lead to substantial mischaracterization of thrombi through a signal processing phenomenon known in the literature as “under-sampling” [39]. On the other hand, it has not been conclusively demonstrated that the standard assessment with two time points is suboptimal when compared to dynamic assessment. Given the inherent trade-off between imaging and time resolution, the possible image resolution for dynamic perviousness is generally lower than that of standard perviousness, which may provide a valid reason to prefer the latter. Moreover, measuring and calculating standard perviousness is considerably easier and faster than dynamic perviousness. Given current methods, this provides a significant advantage for standard assessment in the acute setting of AIS.

Dynamic perviousness can be measured using image sequences from either multiphase CTA or CTP. In general, CTP provides a much higher temporal resolution for CAU sampling compared to multiphase CTA. It captures the CM penetration every 1–3 s over a period of 45–90 s, whereas standard multiphase CTA captures only 3–4 time points at several seconds intervals over a much longer time span. From a signal-processing perspective, the higher the sampling rate of the CAU, the more accurate its characterization becomes [39]. On the other hand, the increased temporal resolution of CTP typically comes at the expense of reduced image resolution, particularly along the slice direction. The ideal temporal (number of image phases) and corresponding spatial resolution remains unknown as no comparative study has yet been conducted to evaluate multiphase CTA versus CTP in assessing perviousness.

Additional factors like symptom onset and medication can also affect the measured values. The longer a clot persists before imaging, the more likely it is to become organized and less pervious. As time passes, the clot may become more fibrin-rich [21], influencing perviousness. Anticoagulants prevent new clots or the growth of existing ones by inhibiting factors in the coagulation cascade [40]. They can influence the size and stability of the clot, indirectly aiding in thrombus perviousness. Antiplatelet (e.g., aspirin, clopidogrel) prevent solid thrombus formation [41] while thrombolytic agents (e.g., tissue plasminogen activator—tPA) dissolve clots [42], both of them potentially influencing perviousness over time.

## Perviousness and composition

The use of MT has enabled the histopathological analysis of the retrieved clots. In most published studies, the general structure of the thrombus was examined using hematoxylin and eosin (H&E) and Martius Scarlet Blue (MSB) staining. H&E staining differentiates between RBCs, fibrin/platelet zones, and WBCs, and is used to quantify each component within the thrombus [10, 22]. MSB staining provides further distinction between fibrin and platelets. After staining, clot slices are analyzed with tools like the Orbit Image Analysis software (Orbit Image Analysis, Idorsia Ltd), and the proportion of each component is calculated [10, 22]. Based on the relative prevalence of these structural components, clots are typically classified as RBC-, WBC-, fibrin or fibrin/platelet-rich.

Thirteen studies examined the link between thrombus perviousness and histopathological composition and there is a wide variation in the reported findings (Table 1). For example Benson et al. [30], Wang et al. [43], Ye et al. [20] and Kim et al. [44] found a positive correlation between perviousness and RBC and an inverse correlation between perviousness and fibrin, while Berndt et al. [45], Patel et al. [46], Gao et al. [47], Ikenberg et al. [48] and Hund et al. [49] reported the opposite and found a positive correlation between fibrin and perviousness and an inverse correlation between RBC and perviousness. In a recent study by Schartz et al. [50] the association between perviousness and the proteomic composition of the removed thrombi was investigated. In addition, the authors conducted an abundance analysis to investigate the difference in protein abundance between pervious and impervious thrombi. Using a sample size of 59 patients, a total of 2790 distinct proteins were identified in the retrieved thrombi, of which 147 were significantly related to perviousness. Pervious thrombi showed a significant depletion of RBCs and iron processing with an abundance of platelet proteins and a significant depletion of RBC hemoglobin. Wei et al. [38] used dynamic perviousness and found that unimodal CAU with one clear peak was associated with fibrin-rich thrombi while a linear CAU showed a strong association with RBC-rich thrombi.

**Table 1:** Studies that examined the link between thrombus perviousness and histopathological composition

Author (Year)	*n*	Method	Components reported	Results
Berndt et al. (2018)[45]	133	TAI_standard_ & void fraction	fibrin/platelet, RBC	Positive correlation with fibrin/platelets conglomerations, inverse correlation with RBC
Benson et al. (2020)[30]	57	TAI_standard_	fibrin, RBC, WBC	Positive correlation with RBC, negative correlation with fibrin Impervious thrombi more likely to be fibrin or WBC-rich
Patel et al. (2021)[46]	40	TAI_standard_	fibrin/platelet, RBC, WBC	Positive correlation with fibrin/platelets aggregates, inverse correlation RBCs
Ye et al. (2021)[20]	53	TAI_standard_	fibrin/platelet, RBC	Positive correlation with RBC fraction, inverse correlation with platelet fraction No correlation with fibrin fraction
Gao et al. (2022)[47]	55	TAI_standard_	fibrin/platelet, RBC, WBC	Positive correlation with fibrin, negative correlation with RBC
Ikenberg et al. (2022)[48]	64	TAI_standard_	fibrin, RBC, WBC	Higher fibrin/platelet content & higher perviousness in cardioembolic vs. non-cardioembolic thrombi
Hund et al. (2022)[49]	332	TAI_standard_	fibrin, RBC	Positive correlation with fibrin, negative correlation with RBC
Wang et al. (2023)[43]	98	TAI_standard_	fibrin/platelet, RBC, WBC	Positive correlation with fibrin, negative correlation with RBC
Kim et al. (2023)[44]	30	TAI_standard_	fibrin/platelet, RBC	Positive correlation with polyhedrocytes and RBCs
Cahalane et al. (2024)[51]	-	TAI_standard_	platelet, RBC	No correlation with RBC, positive correlation with platelet content
Anagnostakou et al. (2024)[52]	39	dynamic, 3-phases	fibrin/platelet, RBC	Thrombi without contrast agent uptake or with contrast agent uptake without corresponding fast washout were more likely to be RBC-rich
Schartz et al. (2024)[50]	59	TAI_standard_	proteomics	Pervious thrombi was associated with: macrophage marker CD14, hemoglobin subunit, proteins involved in platelet cytoskeleton remodeling (tropomyosin a- 3-chain), granule secretion/aggregation (synaptotagmin-like protein 4/FC region receptor II-a)
Wei et al. (2024)[38]	166	dynamic, 21-phases	fibrin/platelet, RBC, WBC	The shape of the contrast agent uptake curve was associated with fibrin and RBC Unimodal curves: fibrin-rich Linear curves: RBC-rich

The composition of thrombi leading to AIS may differ depending on their origin. Several studies reported higher RBC levels in thrombi associated with large artery atherosclerosis in comparison to thrombi of cardiac or unknown origin [23, 24, 53–55], while others showed the opposite and reported higher RBC levels in thrombi of cardiac origin [19, 56, 57]. In addition, several studies did not find any association between composition and etiology [14, 54, 58, 59]. We found only three studies in which the link between perviousness and stroke etiology was analyzed. Kufner et al. [60] measured TAI_standard_ and void fraction in cardioembolic versus noncardioembolic stroke in 75 patients with proximal occlusions of the middle cerebral artery. Using binary logistic regression analysis, increased perviousness was significantly associated with cardioembolic stroke. Interestingly, the same group conducted a very similar study design in 80 patients with acute basilar artery occlusion in which, in contrast to proximal middle cerebral artery occlusions, perviousness was not associated with stroke etiology [61]. Ikenberg et al. [48] reported that cardioembolic thrombi with or without oral anticoagulation had a higher fibrin content and higher perviousness than non-cardioembolic thrombi.

## Perviousness and clinical parameters

Thirty studies examined the link between thrombus perviousness and clinical parameters in AIS (Table 2). The majority of published studies concentrated on the association for stroke severity (NIHSS score: NIH Stroke Scale scores), revascularization results (by TICI scale: Thrombolysis In Cerebral Infarction) or functional outcome (by mRS at 3-months: Modified Rankin Scale). In addition, several studies investigated the link between perviousness and revascularization parameters such as MT duration, number of MT passes, aspiration-first success, effect of intravenous alteplase or distal embolization.

**Table 2:** Studies that examined the link between thrombus perviousness and clinical parameters of AIS

Author (Year)	n	Method	Parameters	Results
Santos et al. (2016)[26]	308	TAI_standard_	recanalization, mRS at 3-months	Pervious thrombi → higher odds for complete recanalization, favorable outcome
Santos et al. (2016)[62]	184	TAI_standard_ & void fraction	TICI, mRS at 3-months	Pervious thrombi → more likely to recanalize, favorable outcome
Borst et al. (2017)[63]	199	TAI_standard_ & void fraction	mRS at 3-months	Pervious thrombi → favorable outcome. Intra-arterial treatment independent of perviousness
Chen et al. (2018)[36]	104	dynamic, 26-phases	mRS at 3-months	Pervious thrombi → favorable outcome
Santos et al. (2018)[34]	233	dynamic, 3-phases	mRS at 3-months	Pervious thrombi → favorable outcome
Alves et al. (2018)[64]	195	TAI_standard_	mRS at 3-months	High perviousness → high collateral score Pervious thrombi → positive factor only if collateral score moderate or high
Byun et al. (2019)[65]	52	dynamic, 3-phases	first-pass success, TICI	No association with first-pass or successful recanalization using stent retrievers
Dutra et al. (2019)[66]	408	TAI_standard_	TICI, dutration of MT, mRS at 3-months	Pervious thrombi → favorable outcome No association with the duration of MT, reperfusion rates
Mokin et al. (2021)[67]	165	TAI_standard_	first-pass success, TICI	Pervious thrombi → predictor of TICI, first pass success in aspiration first approach
Bilgic et al. (2020)[68]	84	TAI_standard_	response to tPA, mRS at 3-months	Pervious thrombi → better response to tPA, favorable outcome
Kyselyova et al. (2021)[69]	106	TAI_standard_	aspiration success, TICI	No relation with aspiration success
Santos et al. (2021)[70]	195	TAI_standard_	mRS at 3-months	Pervious thrombi → favorable outcome
Waqas et al. (2022)[71]	90	TAI_standard_	aspiration-first success, TICI	Pervious thrombi → first pass effect in the aspiration first approach
Kappelhof et al. (2021)[72]	443	TAI_standard_	effect of endovascular treatment, mRS at 3-months	Pervious thrombi → benefit of alteplase, favorable outcome, decreased chance of mortality
Tolhuisen et al. (2021)[73]	245	TAI_standard_	onset-to-imaging times	Not associated with onset-to-imaging time
Berndt et al. (2021)[29]	188	CTA-index, TAI_standard_ void fraction	NIHSS, TICI, mRS at 3-months	Pervious thrombi → low NIHSS at discharge, better TICI, favorable outcome
Terreros et al. (2022)[74]	149	TAI_standard_	NIHSS, MT time metrics, mRS at 3-months	Not associated with clinical parameters
Gao et al. (2022)[47]	55	TAI_standard_	diabetes melitus with and without admission hyperglycemia	Perviousness in patients with admission hyperglycemia lower than without admission hyperglycemia
Pilato et al. (2023)[75]	100	TAI_standard_	distal embolization during MT	Distal embolization → lower perviousness. Perviousness & contact aspiration → protecting factors against distal embolization
Bala et al. (2023)[76]	496	TAI_standard_	distal embolization during MT	Distal vs. no distal embolization: no difference in perviousness
Dai et al. (2023)[77]	86	CTA-index, TAI_standard_ void fraction	Thrombus iodine concentrations, mRS at 3-months	Pervious thrombi → correlation with iodine concentrations and favorable outcome
Shang et al. (2023)[78]	73	TAI_standard_	mRS at 3-months	Pervious thrombi → favorable outcome, profited from the combination of intravenous alteplease and MT
Bala et al. (2023)[79]	520	TAI_standard_	TICI, number of MT passes, MT duration, first-line MT strategy	Not associated with clinical parameters
He et al. (2023)[80]	49	TAI_standard_	MT passes, MT duration	Pervious thrombi → positive correlation with porosity, MT duration and number of MT attempts
Toth et al. (2024)[81]	137	dynamic, 3-phases	TICI, mRS at 3-months	Thrombi with late contrast enhancement → higher revascularization rate, better outcome
Bertalan et al. (2024)[35]	65	TAI_standard_ vs dynamic 3-phases	NIHSS, TICI, number of MT passes	No association between TAI_standard_ and clinical parameters Dynamic perviousness: association with NIHSS, TICI and number of MT passes
Kamepalli et al. (2024)[82]	69	dynamic, 4-phases	TICI, first-pass recanalization	Pervious thrombi → first-pass recanalization
Terreros et al. (2024)[83]	81	TAI_standard_	mRS at 3-months	Pervious thrombi → early recanalization
Mojtahedi et al. (2024)[84]	2153	TAI_standard_	TICI, mRS at 3-months	Pervious thrombi → favorable outcome
Bertalan et al. (2024)[37]	55	TAI_standard_ vs dynamic 30-phases	mRS at 3-months	No association between TAI_standard_ and outcome. Dynamic perviousness: favorable outcome

Studies marked with green found perviousness to be strongly associated with better recanalization rates and/or favorable outcome

### Perviousness and clinical outcome

Santos et al. [62] found that pervious thrombi were 2.5 and 3.5 times more likely to recanalize and have a favorable outcome, respectively. Using dynamic perviousness and 26-phase dynamic CTA, Chen et al. [36] could predict good clinical outcomes with a 0.73 AOC under the ROC. Using a similar method with 30-phase CTP and time-dependent parameters of the CAU, Bertalan et al. reported the same association between perviousness and clinical outcome [37]. In a study by Biglic et al. [68] and Kappelhof et al. [72], thrombi with higher perviousness responded significantly better to alteplase and had better outcome than thrombi with relatively low perviousness. Pilato et al. [75] found elevated perviousness as a protective factor against distal embolization. The majority of published studies reported similar results and found that increased perviousness was associated with better functional outcome after the intervention (Table 2) [26, 29, 34, 36, 62, 63, 66, 70, 72, 77, 78, 84]. However, some studies also reported contradictory results and found no association between standard perviousness and clinical outcome [35, 37, 74, 76, 79, 83]. In a cohort of 195 patients in Alves et al. [64], pervious thrombi had a positive effect on clinical outcome only if the collateral score was moderate or high. Terreros et al. [83] and Bala et al. [76] used standard perviousness and found no association for functional outcome. Bertalan et al. [35] reported the same result using TAI_standard_ and full 3D volumetric evaluation of thrombi in 65 AIS patients with HAS.

### Perviousness and revascularization parameters

Similarly as for functional outcome, several studies reported an association between perviousness and revascularization parameters [26, 29, 62, 67, 68, 71] while others did not find any association [35, 63, 65, 66, 69, 73, 76, 79, 83, 84]. Using 308 AIS patients, Santos et al. [26] found higher odds for complete recanalization for pervious thrombi. Mokin et al. [67] and Waqas et al. [71] reported that perviousness was a predictor for TICI and high perviousness was associated with first-pass success in the aspiration-first approach. Kapelhoff et al. [72] reported an increased effect of alteplase for pervious thrombi. He et al. [80] studied the link between thrombus porosity, perviousness and MT procedure metrics. They reported a significant negative correlation between non-porous thrombi, perviousness, MT duration and number of MT attempts. Contrary to these studies, Borst et al. [63] found an independent effect of intravenous treatment from perviousness while Byun et al. [65] were not able to predict first-pass recanalization or successful recanalization using stent retrievers with perviousness. Dutra et al. [66] observed no association between the duration of MT or reperfusion rates and perviousness and Kiselyova et al. [69] found no relation between perviousness and aspiration success.

## Discussion

### Are thrombus structural and mechanical properties clinically relevant?

Thrombus composition has been shown to be linked to MT procedure metrics, recanalization, and clinical outcomes [21]. In vitro studies have demonstrated that while some MT devices effectively extract specific thrombus types, they are less successful with others. For example, aspiration is more successful with soft clots, whereas extraction of harder clots may be more efficient with stent-retrievers [85, 86]. The ability of the thrombus to reply to the applied forces during its integration into the stent or during aspirating into a large lumen catheter are determined by its mechanical properties [13, 21], which is largely influenced by its histopathological composition [21, 51]. RBC-rich thrombi have been associated with shorter procedure times, fewer device passes [15, 24] and higher rates of successful recanalization [14, 19, 87]. On the other hand, fibrin-rich human thrombi are less likely to fragment and migrate less easily than RBC-rich thrombi, which is a positive factor during MT [85, 86, 88]. These findings highlight the clinical importance of determining thrombus structural and/or mechanical properties before MT.

### Is it feasible to assess compositional properties with perviousness?

Evaluating clot composition based on perviousness is challenging, which applies to all CT imaging markers [21]. We are uncertain whether the conflicting results in the literature are due to differences in histological methods, variations in imaging protocols and evaluation techniques, or a combination of all.

The majority of studies concentrated on the link between perviousness and fibrin/RBC ratio in the clot (Table 1). However, considering only the RBC/fibrin ratio while disregarding the structural relationship between these components may lead to mischaracterization. Each histopathological building block has different physical characteristics, e.g. viscosity, elasticity, permeability and adhesion. RBCs are less stiff than platelets and are deformable [13, 89, 90]. RBCs rich clots might be pervious if RBCs are present in a loose core, but platelet-driven forces during thrombus maturation can compact them into impermeable layers with elevated stiffness and decreased perviousness [91–93]. Clot contraction results in tightly packed RBCs (polyhedrocytes), which can decrease the perviousness [91].

**Fig. 4:**
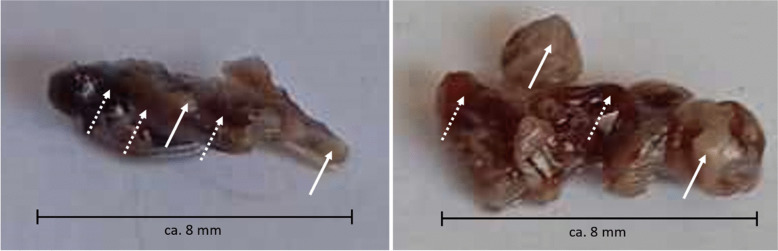
Retrieved clots from two different patients (left: 66-years-old woman, clot from internal carotid artery; right: 78-years-old man, clot from middle cerebral artery). Arrows illustrate visually noticeable sub-regional differences in tissue structure. Areas marked with dotted arrows have probably relatively high RBC and low fibrin content as indicated by red-colored tissue, while areas marked with solid arrows have probably relatively high fibrin and low RBC content as indicated by white-colored tissue. Both example clots demonstrate, that thrombus can have RBC-dominant and fibrin-dominant sub-volumes in the same sample and, therefore, the histopathological classification of the clots as RBC-rich or fibrin-rich is challenging

There is no universally accepted method for classifying thrombi based on their RBC/fibrin ratio, as different groups apply varying methods and thresholds for their classifications. Furthermore, inter-operator variability in histopathological analysis remains a significant challenge due to the complexity of accurately sampling the tissue for analysis. Histology analyzes only thin sections, typically a few micrometers in thickness, and the heterogeneity of thrombus (Fig. 4) may introduce potential sampling bias into the analysis, significantly affecting the consistency of the results.

Histology operates on the micrometer scale, whereas CT imaging operates on the millimeter or sub-millimeter scale. The spatial registration of histological findings to imaging data is therefore challenging, particularly in the context of clot imaging, where the clot is often not fully visible on CT scans. Variations in histology methods (e.g., MSB vs. H&E staining), sample preparation and evaluation may have also played a role in the conflicting results.

Standardization is required in the histological characterization of thrombi to support multicenter studies. Furthermore, in addition to the traditional RBC/fibrin ratio, a more detailed analysis of the fibrin and RBC matrix is essential, as it plays a crucial role in the mechanical properties and behavior of thrombi during MT [21]. Advanced proteomic analyses could also contribute to enhancing our understanding of thrombus biology [50]. Such approaches could help clarify discrepancies between histological composition and perviousness by providing a more detailed characterization of clot components.

Even if standardization is achieved and advanced histological methodologies for clot characterization are applied, several challenges will remain in assessing clot composition through perviousness. For instance, the longer a clot has been in place, the greater the likelihood it will become organized, potentially impacting its perviousness with little to no change in its histological composition [21]. In our opinion, these difficulties motivate shifting the focus from histological structure to overall mechanical characterization with perviousness. The interventionalist seeks to ascertain whether a clot is soft, stiff, or prone to fragmentation. This information is crucial for determining the optimal removal strategy. Whether a thrombus is RBC- or fibrin-rich is less important in this regard. The mechanical properties can be evaluated on the same scale as perviousness and seem to be more readily assessed through imaging than the clot’s structural characteristics. Unfortunately, we did not find any studies that investigated the association between mechanics and perviousness, despite the urgent need for such research.

### Is thrombus perviousness clinically relevant?

Table 2 demonstrates the balance between conflicting results regarding the association between perviousness and clinical parameters. The majority of published studies indicate that increased clot perviousness is strongly linked to improved recanalization rates and/or favorable outcomes. There are multiple potential explanations for this. In most cases, MT is performed alongside intravenous thrombolysis, which can enhance the success rate of reperfusion. From a physical standpoint, a porous and loosely packed structure is essential for the thrombolytic drug to penetrate deeply into the thrombus and exert its effect. This assumption was confirmed by several studies that showed that the benefit of alteplase increased with more pervious thrombi [68, 72, 78, 80]. It has also been suggested that the high perviousness of the clot allows residual blood flow through the blocked artery, at least partly, potentially reducing tissue damage and extending the time window for MT. Additionally, it is assumed that perviousness is inherently related to the tissue density of the clot. Several studies reported that intervention times of MT for soft clots were shorter and a smaller number of MT attempts was required [14–16, 85], while harder clots were generally more difficult to remove [11, 13, 54, 94, 95]. This highlights the need for a direct correlation between perviousness and tissue mechanics, which is currently underexplored in the literature.

The small number of contradictory findings regarding perviousness and clinical parameters may be attributed to the used measurement methods. In all these studies, perviousness was calculated within spherical ROIs on a 2D transverse slice using TAI_standard_, which can lead to mischaracterization as described in Section “Measurement of thrombus perviousness with CT” In a study by Bertalan et al. [35], using full 3D volumetric segmentation and only thrombi with HAS, no correlation was observed for TAI_standard_, while dynamic perviousness showed a significant correlation with TICI and the number of MT passes. Additional studies are required using multicenter datasets and dynamic perviousness with standardized protocols to resolve these controversies.

The numerous contradictory findings regarding recanalization parameters make it difficult to assess the benefit of perviousness in MT strategy planning. We found six studies that indicated a link with MT procedure metrics [26, 29, 62, 67, 68, 71] and ten studies that found no association [35, 63, 65, 66, 69, 73, 76, 79, 83, 84]. The MT metrics utilized in these studies, including first-pass success, aspiration-first approach and duration of MT, are affected by various human factors, such as the experience of the interventional team and the patient’s health condition. We are unsure if the conflicting results are caused by human factors, differences in imaging protocols and evaluation techniques, or a combination of these. Additional studies are required to resolve these controversies. In vitro MT utilizing vascular models that replicate real anatomy under physiological hemodynamic conditions, along with clot analogs derived from human or animal blood, can address this gap [55, 96, 97] and determine the role of perviousness in MT method selection.

### Main barriers to integrating perviousness assessment into clinical practice

The utilization of standard CT protocols is unquestionably an advantage of perviousness. The methodological background seems to be feasible in the acute settings of AIS. However, along with addressing the reported controversies surrounding the relationship to structure, clinical, and MT procedure metrics, several other obstacles must be overcome before perviousness can be incorporated into the clinical practice. This includes the standardization of image acquisition protocols, assessment, segmentation and large multicenter trials with standardized methods for validating its performance.

A key factor contributing to variation in the TAI_standard_ is the time interval between CM injection and CTA acquisition. As the CM bolus first passes through the regional circulation, it remains within the vascular space and the CM uptake into the thrombus volume is mainly driven by penetration and diffusion through its surface and outer shell. Since the wash-in and wash-out time in the vascular space is relatively rapid, the available time window for CM uptake by the thrombus is relatively short. In [37], the authors used a 30-phase CTP acquisition and showed, that for TAI_standard_, the CTA acquisition has to hit a 14 ± 4 s time window to capture the CAU within 30% of its arterial peak. This is technically very challenging and emphasizes the importance of utilizing a dynamic assessment of perviousness. Chen et al. came to the same conclusion using 26-phase CTP [36]. Unfortunately, only a small number of studies used dynamic perviousness with more than four time points [36, 37, 82].

The workflow of thrombus segmentation and corresponding quantitative evaluation needs to be optimized. In the acute setting of AIS, evaluation has to be feasible within minutes. In research image analysis software such as 3D Slicer [98], a full 3D manual segmentation can be done within 5 min, which seems to be feasible in the clinical routine. After image registration and segmentation, perviousness can be rapidly computed within seconds using computer algorithms. Unfortunately, there is a shortage of specialized software that integrates both segmentation and perviousness calculation into a single, streamlined tool designed for clot characterization—an essential step for incorporating perviousness into clinical practice. Recently, AI-driven methods have been under development and are showing promising results in automatically segmenting thrombi on standard CT images [84, 99, 100] —one of the key prerequisites for automated evaluation software.

### What else is needed?

Dynamic perviousness represents an important step toward improved thrombus characterization. However, it uses standard protocols, which either have high through-plane, in-plane- or temporal-resolution, but not all three at the same time. Therefore, there is a need for dynamic time-resolved protocols that capture the CM penetration in the thrombus with a relatively high signal-to-noise ratio at relatively high 3D and temporal resolution. Dual-energy or photon counting CT, with its improved resolution and image quality compared to conventional CT, could play an important role here.

CT protocol parameters should also be standardized, at least to the extent possible. For instance, there is significant variability among clinics in the used CM dose and time delays between NCCT and post-contrast CT (CTA or CTP) acquisition. These discrepancies can significantly impact the measured values. While standardization is inherently challenging due to differences in scanner types and manufacturers, it is essential to make efforts to reduce these variations between clinical sites to ensure the comparability of results.

Thrombus calcification refers to the presence of calcium deposits within a clot, typically appearing as high-density areas and contributing to the HAS on NCCT [51]. This finding often indicates an organized thrombus and is generally linked to poor clinical outcomes or resistance to thrombolytic therapy [22]. Assessing clot perviousness may improve the detection of calcified thrombi, though this area remains relatively underexplored in current research.

More research is needed to address the role of MRI in clot characterization. Although the relative benefit of MRI in comparison with CT is debated, there is a shift from CT to MRI in AIS evaluation in certain countries [101]. Unfortunately, clot evaluation with MRI is underdeveloped and imaging markers as well as protocols are still to be defined.

## Conclusion

Information on thrombus structural properties before the intervention could help to optimize the revascularization strategy. Thrombus perviousness is a promising imaging biomarker for thrombus structural characterization and has been associated with several clinical parameters including reperfusion rates and functional outcome. Currently, increased perviousness seems to be a positive prognostic factor of functional outcome after the intervention, although some conflicting results have been also reported. Dynamic perviousness is a recently developed extension of the classical standard method that could further increase its role in the diagnosis. To effectively incorporate perviousness into clinical practice, the conflicting results related to thrombus structure and thrombectomy parameters as well as the barriers on the technical side must be resolved. In addition, the focus of radiological imaging may need to shift from structure to mechanical characterization of the clot to predict device-tissue interaction.

## Data Availability

No datasets were generated or analysed during the current study.

## Abbreviations

AIS: Acute ischemic stroke
MT: Mechanical thrombectomy
RBC: Red blood cell
WBC: White blood cell
CT: Computed tomography
CM: Contrast material
NCCT: Non contrast CT
CTA: CT angiography
CTV: Late venous phase CT
CTP: CT perfusion
TAI: Thrombus attenuation increase
HU: Hounsfield unit
ROI: Region of interest
TAI_standard_: Standard perviousness measured with two imaging time points
CAU: Time resolved CM uptake curve in thrombi
